# Integrated Methylome and Transcriptome Analysis between Wizened and Normal Flower Buds in *Pyrus pyrifolia* Cultivar ‘Sucui 1’

**DOI:** 10.3390/ijms25137180

**Published:** 2024-06-29

**Authors:** Hui Li, Chunxiao Liu, Jialiang Kan, Jin Lin, Xiaogang Li

**Affiliations:** Institute of Pomology, Jiangsu Academy of Agricultural Sciences, Nanjing 210014, China; lihui7904@163.com (H.L.); lcx@jaas.ac.cn (C.L.); 201800701@jaas.ac.cn (J.K.); lj84390224@126.com (J.L.)

**Keywords:** pear, wizened bud, methylation feature, auxin, cytokinin

## Abstract

Here, cytosine methylation in the whole genome of pear flower buds was mapped at a single-base resolution. There was 19.4% methylation across all sequenced C sites in the *Pyrus pyrifolia* cultivar ‘Sucui 1’ flower bud genome. Meantime, the CG, CHG, and CHH sequence contexts (where H = A, T or C) exhibited 47.4%, 33.3%, and 11.9% methylation, respectively. Methylation in different gene regions was revealed through combining methylome and transcriptome analysis, which presented various transcription trends. Genes with methylated promoters exhibited lower expression levels than genes with non-methylated promoters, while body-methylated genes displayed an obvious negative correlation with their transcription levels. The methylation profiles of auxin- and cytokinin-related genes were estimated. And some of them proved to be hypomethylated, with increased transcription levels, in wizened buds. More specifically, the expression of the genes *PRXP73*, *CYP749A22,* and *CYP82A3* was upregulated as a result of methylation changes in their promoters. Finally, auxin and cytokinin concentrations were higher in wizened flower buds than in normal buds. The exogenous application of paclobutrazol (PP333) in the field influenced the DNA methylation status of some genes and changed their expression level, reducing the proportion of wizened flower buds in a concentration-dependent manner. Overall, our results demonstrated the relationship between DNA methylation and gene expression in wizened flower buds of *P. pyrifolia* cultivar ‘Sucui 1’, which was associated with changes in auxin and cytokinin concentrations.

## 1. Introduction

Pear (*Pyrus* spp.), as the third most important temperate fruit genus, is widely cultivated throughout the world [1]. In China, different pear species, such as *Pyrus pyrifolia*, *Pyrus bretschneideri*, *Pyrus ussuriensis*, *Pyrus sinkiangensis,* and *Pyrus communis*, are distributed in different geographical regions and their popularity is exploited for specialty fruit production [2]. However, the phenomenon of wizened buds is frequently observed in pear trees, making it one of the crucial factors hindering further pear development. Currently, some pear varieties exhibit a high proportion of wizened floral buds [3,4], which results in considerable decreases in crop yield [3,4,5]. Previous studies had demonstrated that wizened bud formation was strongly associated with management practices and the nutritional status of the buds [5]. As with other crops, physiological and transcriptomic differences occur in association with the formation of wizened buds in pear [3]. For instance, transcription changes in some genes related to antioxidant enzyme activities or changes in hormone concentrations were associated with wizened buds. Nevertheless, the intrinsic molecular mechanisms by which wizened buds in pear are formed remain uncharacterized.

Many Rosaceae fruit trees, such as pear, apple, and peach, display similar wizened bud morphologies [3,4,5,6,7]. These abnormal buds usually form during the later stages of bud differentiation and have loose external scales and brown internal bud organs, developing non-functional flowers. Their formation is due to the inhibition of the bud differentiation process for various reasons. Usually, the balance between meristem size and the initiation and coordination of organs is the foundation of flower development. During this process, hormone balance plays an essential role. Firstly, cytokinins, gibberellins, and auxin work together to correct the floral meristem size, and then auxin regulates organ initiation and organogenesis [8]. Once the balance of endogenous hormones in the bud is disturbed, flower bud development stops and wizened buds may develop. Investigation of wizened buds in pear found that the concentrations of cytokinins and auxin increased and the ratios of different hormones changed in these abnormal buds [4,5]. We hypothesize that the incidence of wizened buds could decrease as a result of regulating plant hormone metabolism and rebuilding the balance between vegetative growth and reproductive growth.

Development of flower buds is associated with the regulation of gene expression in plants. Several lines of evidence also suggest relationships between the status of flower buds and phytohormone concentrations and ratios [5,8,9]. Indeed, transcriptome analysis for different types of flower buds from pear revealed changes in auxin metabolism, transport, and signal transduction pathways [10,11]. The formation of wizened buds in pear could be triggered by ambient environmental stresses that influence expression levels of hormone biosynthesis genes [5]. Plants have evolved several strategies, such as epigenetic modifications, to respond to environmental stresses [12,13]. Gene expression regulates various developmental and physiological processes through the methylation of DNA [14]. In other words, epigenetics-based DNA methylation coordinates stress-regulated flowering via gene expression patterns [13]. But, the relationship between DNA methylation and gene expression still needs to be investigated to reveal when flower development stops and wizened bud formation starts.

In the current study, ‘Sucui 1’ sand pear (*Pyrus pyrifolia*) plants were chosen as the research material, with typical wizened buds occurring during the growing season. The physiological changes associated with wizened bud formation were determined through microscopic observations and phytohormone analyses. Moreover, RNA-Seq analyses and whole-genome bisulfite sequencing (WGBS) were used to conduct the transcriptomic and methylome comparative profiling of wizened and normal flower buds. Finally, the possibility of exogenous hormones reducing the incidence of wizened flower buds was explored. The aim of this study was to investigate the possible mechanisms underlying wizened flower bud formation in order to provide a new strategy to reduce or even eliminate wizened flower bud occurrence in pear trees.

## 2. Results

### 2.1. Methylation Patterns of Pear Flower Buds

**Figure 1:**
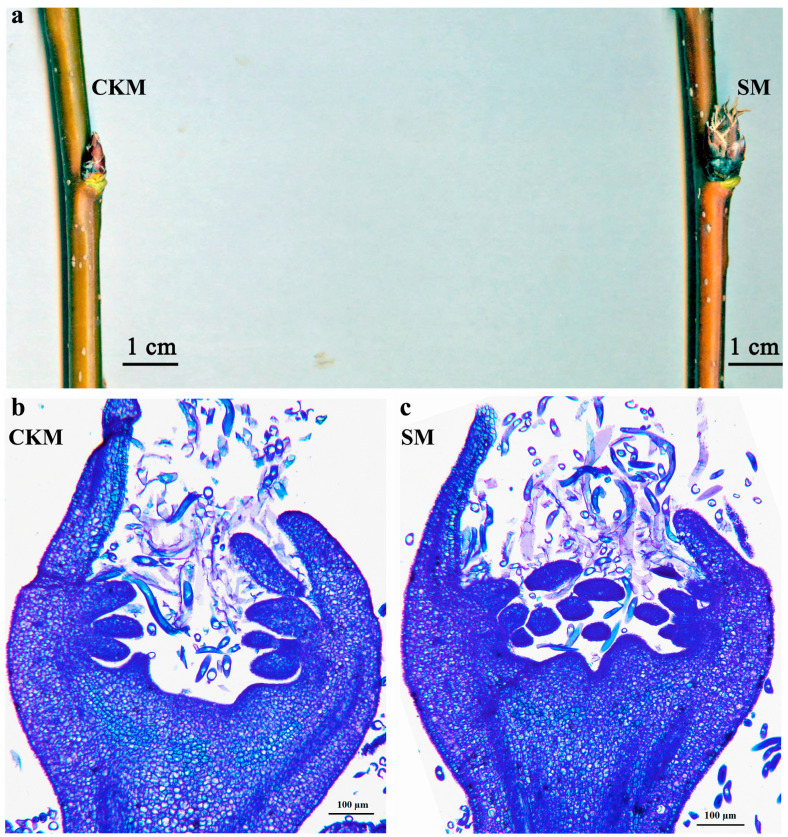
Phenotypic comparison of the normal flower bud (CKM) and the wizened flower bud (SM) of *Pyrus pyrifolia* ‘Sucui 1’. (**a**) Representative phenotypes of the normal flower bud and the wizened flower bud collected on 15 September 2022. (**b**,**c**) Paraffin sections of the normal flower bud (CKM, (**b**)) and the wizened flower bud (SM, (**c**)), stained with 1% toluidine blue, which were collected on 15 September 2022.

The typical phenotypes of the normal flower buds (CKM) and the wizened flower buds (SM) from *P. pyrifolia* cultivar ‘Sucui 1’ and the corresponding paraffin sections stained with 1% toluidine blue are shown in Figure 1. The scales of SM buds loosened, and their tips became grayish brown and wizened, whereas CKM buds remained compact and reddish brown in color (Figure 1a). The stamens of CKM buds had a typical morphology (Figure 1b), whereas the stamens in SM buds aborted and degenerated (Figure 1c).

**Figure 2:**
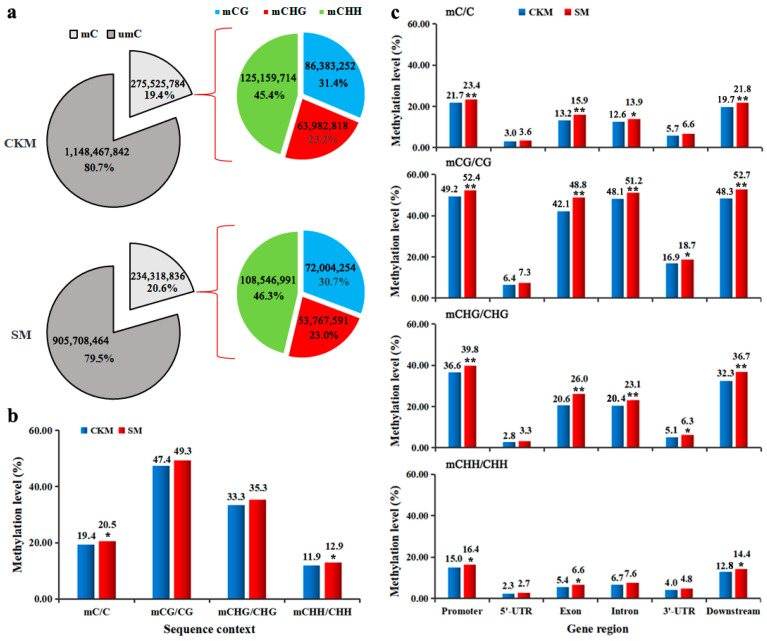
Comparative analysis of DNA methylation patterns between the normal flower bud (CKM) and the wizened flower bud (SM). (**a**) Relative proportions of methylated cytosines (^m^Cs) in each sequence context. (**b**) Statistics of methylation levels (MLs) in each sequence context across the whole pear genome. (**c**) Average MLs in different gene regions. The SPSS26 was used for statistical analysis. Student’s *t*-test was used for comparing two samples, asterisks indicate statistically significant differences between normal (CKM) and wizened (SM) flower buds (* shows *p* < 0.05 and ** means *p* < 0.01).

To investigate the role of DNA methylation dynamics in flower bud development, the whole-genome bisulfite sequencing (WGBS) technique, operating at single-base resolution with high confidence, was used to decipher cytosine methylation across the entire genomes of CKM and SM flower buds (Figure 2a). Supplementary Materials, Table S1, show the results of the WGBS study. The Q30 percentages of each sample exceeded 88%, and the total genome data generated was 73.6 Gb and 61.1 Gb for CKM and SM, respectively, which contained more than 200 million 150 bp paired-end raw reads. Both of the sample genomes represented >38× of the pear reference genome, where the genome size was about 532.7 Mb [15] (Supplementary Materials, Table S1). Then, adapters were trimmed and low-quality reads were filtered, obtaining a total of 239,775,951 and 199,502,834 clean reads for CKM and SM flower buds, respectively. The mapping rates of unique cytosines (C) in CKM and SM were 38.3% and 37.2%, respectively. These mapped data were then used to retrieve the methylation level (ML) of each cytosine site in the CG, CHG, and CHH sequence contexts.

We obtained more than 1140 million cytosines from each sample for further analysis (Figure 2a). Among them, a total of 275,525,784 potentially methylated cytosine sites (mCs) (31.3% at CG sites, 23.2% at CHG sites, 45.4% at CHH sites, with H representing A, T, or C) in CKM samples and 234,318,836 ^m^Cs (30.7% at CG sites, 23.0% at CHG sites, and 46.3% at CHH sites) in SM, respectively, were obtained (Figure 2a). In addition, the percentages of ^m^C, ^m^CG, ^m^CHG, and ^m^CHH in the corresponding cytosine contexts were obtained across the whole genome of CKM and SM flower buds. We found the MLs in CG, CHG, and CHH contexts presented similar comparative trends between the two samples, although the overall degree of genomic methylation of ^m^Cs was significantly higher in SM (20.5%) than in CKM (19.4%) (Figure 2a). When the percentages of DNA MLs in C, CG, CHG, and CHH contexts were determined across the 17 pear chromosomes, the results revealed that the hypermethylation in each chromosome in SM, compared with CKM, was due to increased methylation at CHH, CG, CHG, and CHH sites (Supplementary Materials, Figure S1). To further compare the DNA methylation profiles of the two samples in various gene regions, the methylation patterns were analyzed in the promoter, 5’-UTR (untranslated region), exons, introns, 3’-UTR, and downstream sequences. For C, CG, CHG, and CHH contexts, the MLs within the promoter, exons, introns, 3’-UTR, and downstream regions were significantly higher in SM than in CKM (Figure 2c). Remarkably, in both CKM and SM the CG context exhibited the highest ML, while the CHH context exhibited the lowest ML among the three contexts in all gene regions (Figure 2c).

### 2.2. Analysis of Differential Methylation between CKM and SM

**Figure 3:**
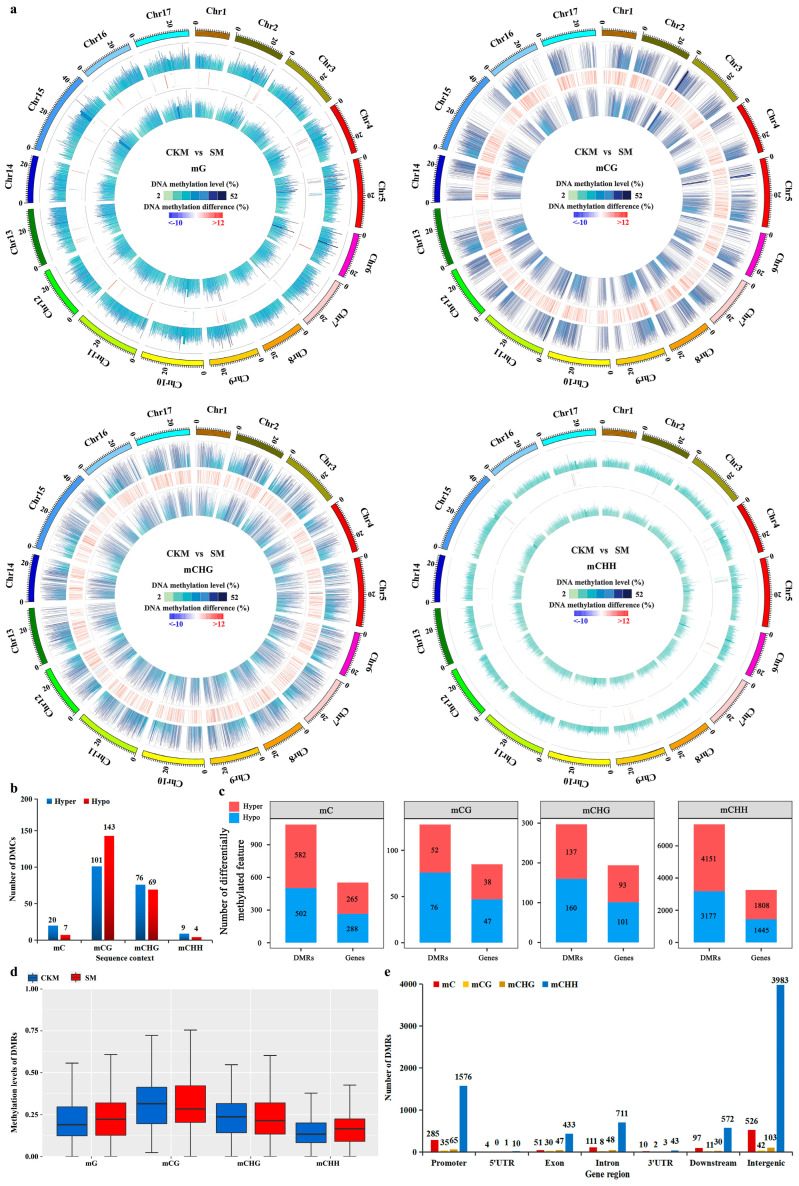
Differential methylome analysis between the normal flower bud (CKM) and the wizened flower bud (SM). (**a**) Circos plot showing the difference in overall MLs between the two samples. The outermost rim indicates the chromosome name and scale. The other tracks from outside to inside represent the following: MLs in CKM or SM buds and the difference in overall MLs in CKM versus SM buds. (**b**) Numbers of differentially methylated cytosines (DMCs) in SM relative to CKM are shown for the ^m^CG, ^m^CHG, and ^m^CHH sequence contexts. (**c**) Numbers of differentially methylated regions (DMRs) and differentially methylated genes (DMGs) in SM relative to CKM buds. (**d**) Box plot of the MLs of differentially methylated regions (DMRs) between CKM and SM buds. (**e**) The distribution statistics of the functional gene regions associated with DMRs.

Quantitative analysis of the differentially methylated cytosines (DMCs) and regions (DMRs) allowed for the characterization of DNA methylation differences between the CKM and SM samples. Four Circos plots were used to display the differences in overall MLs between the two samples, revealing particularly widespread DNA methylation increases in SM (Figure 3a). A total of 402 DMCs were identified in SM relative to CKM, 53.7% and 46.3% of which were hypomethylated (hypo-DMCs) or hypermethylated (hyper-DMCs), respectively. It is noteworthy that 54.3% (the proportion of 101 to the sum of 101, 76, and 9) of the hyper-DMCs occurred in the CG sequence context, implicating an increase mainly in CG DNA methylation in SM relative to CKM (Figure 3b).

To better understand the changes in DNA methylation between CKM and SM samples, the DMRs between their methylomes were identified through Fisher’s exact probability test [16]. A total of 7753 DMRs were identified, of which the average length was largely found to be approximately 100 bp (Supplementary Materials, Figure S2). The genes located in DMRs, the so-called differentially methylated genes (DMGs), were also characterized. In total, 3532 DMGs were identified in SM compared to CKM, of which 3413 hypomethylated DMRs overlapped with 1593 hypomethylated genes and 4340 hypermethylated DMRs containing 1939 hypermethylated genes (Figure 3c). We found that the overall ML of DMRs in SM was slightly higher than that of CKM (Figure 3d). Furthermore, the distribution characteristics of the functional gene regions connected with DMRs were also compared between the CKM and SM samples (Figure 3e), with the promoter regions containing the most hypomethylated and hypermethylated DMRs.

### 2.3. Differences in Changes in DNA Methylation Patterns and Gene Expression Levels during Flower Bud Wizening

**Figure 4:**
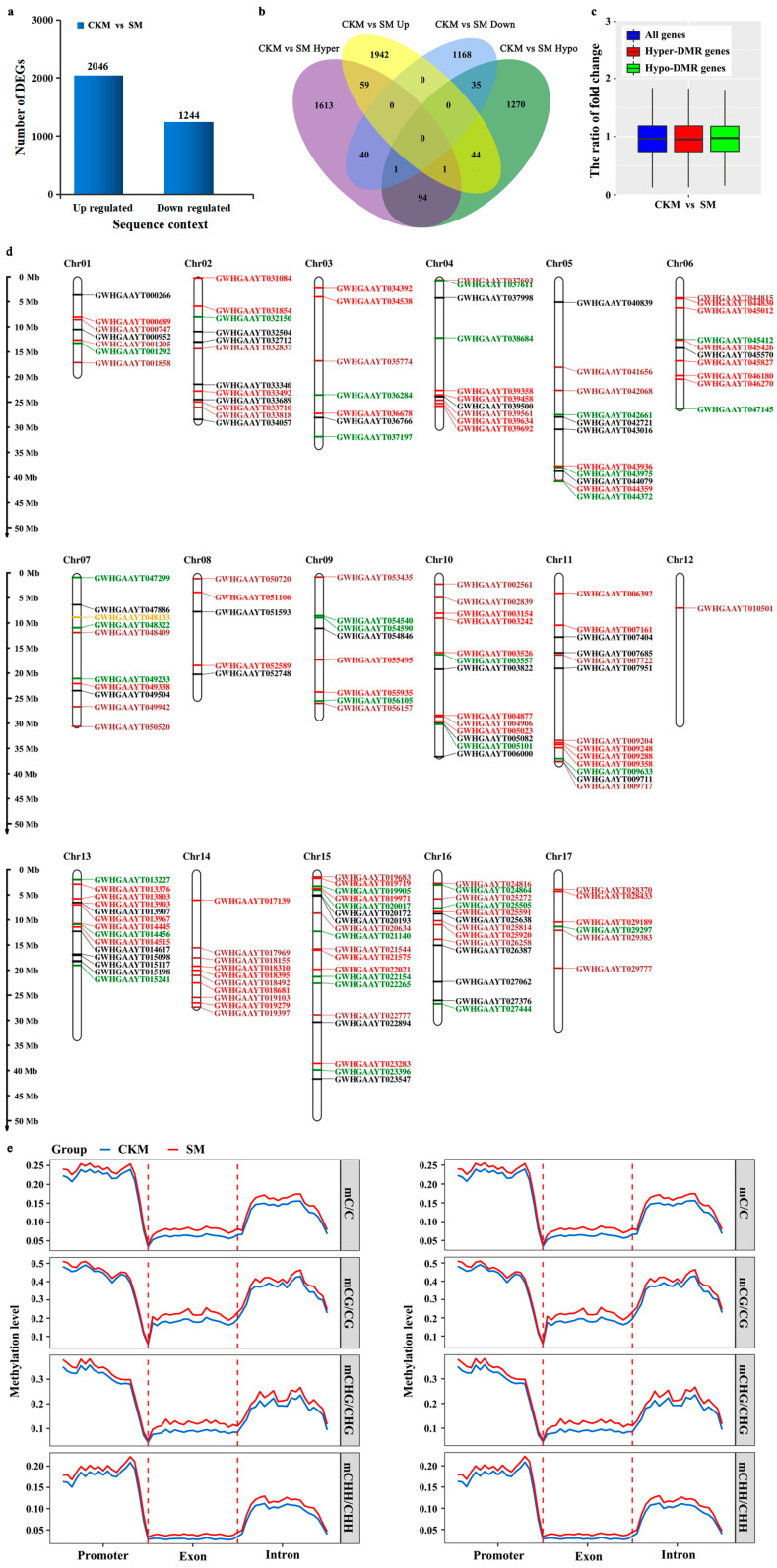
Correlations between altered DNA methylation patterns and differential gene expression. (**a**) Numbers of differentially expressed genes (DEGs) in normal (CKM) relative to wizened (SM) flower buds. (**b**) Venn diagram showing overlaps between the differentially methylated genes (DMGs) and DEGs for the CHH sequence context. (**c**) Boxplot showing the differential expression levels of all genes and hyper− and hypomethylated genes. (**d**) Location distribution of DEGs associated with differentially methylated regions (DMEGs) for the CHH sequence context on different chromosomes of pear. The red and black colors represent upregulated DEGs associated with only hyper− and hypomethylated DMRs, respectively. The purple and green colors represent downregulated DEGs associated with only hyper− and hypomethylated DMRs, respectively. The orange color represents downregulated DEGs associated with both hyper-and hypomethylated DMRs. (**e**) Comparative analysis of MLs of C−sites in different gene regions of the up− or downregulated DEGs, including promoter, exon, and intron regions.

Transcriptome profiles were generated, with three biological replicates of each sample, and the information generation was used for methylome analysis, to determine whether changes in DNA methylation were associated with gene expression changes during the development of wizened flower buds. Principal component analysis (PCA) was employed to visualize and evaluate the overall differences in gene expression between two flower buds. According to the PCA, the three replicates of each sample were closely gathered together, indicating a high consistency and quality of the data (Supplementary Materials, Figure S3). PC1 and PC2 explained 60.4% of the variation observed, and the first PC (41.1%) separated two type flower buds. Our results indicated relatively high positive correlations between the three replicates of the same sample in this study, a finding which supported the accuracy and reliability of the subsequent quantitative gene expression analysis. Additionally, we identified differentially expressed genes (DEGs), with 1244 downregulated and 2046 upregulated DEGs identified in SM compared to CKM (Figure 4a and Supplementary Materials, Figure S4).

Firstly, to gain insight into the possible association between changes in DNA methylation and gene expression, the overlap between the DMGs and DEGs was checked. Forty genes were hypermethylated with downregulated expression levels, and forty-four genes were hypomethylated with upregulated expression levels in SM compared to CKM flower buds. In addition, 35 downregulated and 59 upregulated genes were hypomethylated and hypermethylated, respectively, in SM compared to CKM (Figure 4b).

Secondly, the differential expression levels of all genes and genes located in hyper- or hypomethylated DMRs were comparatively analyzed, with the results presented as a boxplot. Unexpectedly, hypomethylated genes (green box) exhibited a slightly lower ratio of fold change compared with hypermethylated genes (red box) and all genes (blue box) (Figure 4c). In Figure 4d (showing only the ^m^CHH sequence context), a total of 187 non-redundant DEGs associated with DMRs (DMEGs) were identified, namely 176 that were widely distributed across the 17 chromosomes of pear flower buds and 11 DMEGs on scaffolds and contigs. Comparative analysis was also carried out on MLs and the densities of C-sites in the CG, CHG, and CHH contexts in different gene regions of the up- or downregulated DEGs, namely the promoter, exon, and intron regions (Figure 4e and Supplementary Materials, Figure S5). The three gene regions of the upregulated and downregulated DEGs displayed significantly higher MLs in the CG, CHG, and CHH contexts in SM relative to CKM flower buds (Figure 4e).

### 2.4. KEGG and GO Enrichment Analysis of DMEGs

To further investigate the potential role of DNA methylation changes in pear flower buds once wizening had occurred, a Gene Ontology (GO) analysis of DMEGs was first carried out. We found 68 hypermethylated DEGs (hyper-DEGs) in SM compared to CKM, which were annotated to 180 functional categories, namely 116 biological processes (BPs), 11 cellular components (CCs), and 53 molecular functions (MFs) (Supplementary Materials, Table S2). Here, BPs related to “trehalose biosynthetic process”, and “xylem development” were important functional GO terms involved. In addition, the CCs included terms such as “vesicle”, “filiform apparatus”, and “nuclear body”, while “peroxidase activity”, “transferase activity, transferring hexosyl groups”, and “phosphatase activity” were the three most significantly enriched MF terms (Supplementary Materials, Figure S6a). The 62 hypomethylated DEGs (hypo-DEGs) were annotated to 186 functional categories, namely 116 BPs, 19 CCs, and 50 MFs (Supplementary Materials, Table S2), of which the highest enrichment ratios belonged to the BP term “root meristem growth” and the CC term “plasmodesmatal endoplasmic reticulum” (Supplementary Materials, Figure S6b).

**Figure 5:**
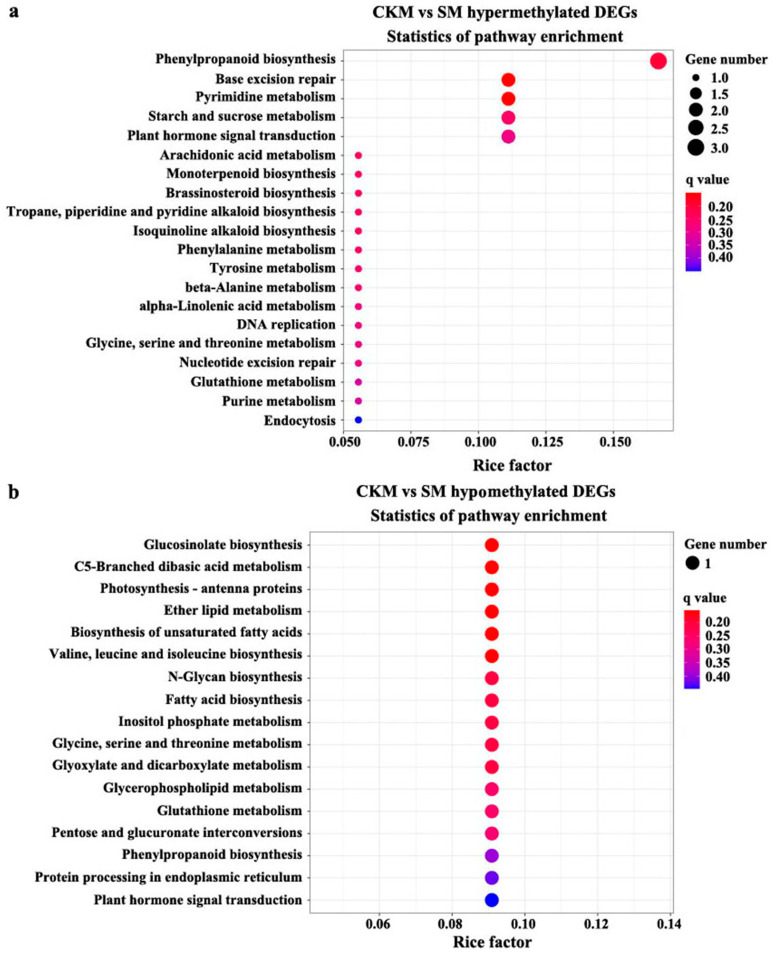
Kyoto Encyclopedia of Genes and Genomes (KEGG) pathway enrichment analysis of hyper-(**a**) or hypomethylated (**b**) DEGs for the CHH sequence context in normal (CKM) versus wizened (SM) flower buds. The gene ratio is equal to the proportion of the number of differentially methylated genes (DMGs) annotated in the pathway to the total number of DMGs. The size of the circle represents the number of genes, and the color of the circle represents the *q*-value (corrected *p*-value).

To better understand the biological functions of DMEGs in SM compared to CKM, a Kyoto Encyclopedia of Genes and Genomes (KEGG) pathway enrichment analysis was performed. The hyper-DEGs in SM compared to CKM flower buds were mainly assigned to pathways related to “base excision repair”, “pyrimidine metabolism”, “phenylpropanoid biosynthesis”, and “arachidonic acid metabolism” (Figure 5a and Supplementary Materials, Table S3), whereas, among the hypo-DEGs in SM compared to CKM, “glucosinolate biosynthesis” was the most enriched pathway, followed by “C5-branched dibasic acid metabolism”, and “photosynthesis-antenna proteins”. The first two pathways were also the most significantly enriched pathways in SM compared to CKM, with a corrected *p*-value < 0.05 (Figure 5b and Supplementary Materials, Table S3).

### 2.5. Association between DNA Methylation, Gene Expression and Hormone Homeostasis to Avoid Bud Wizening

**Figure 6:**
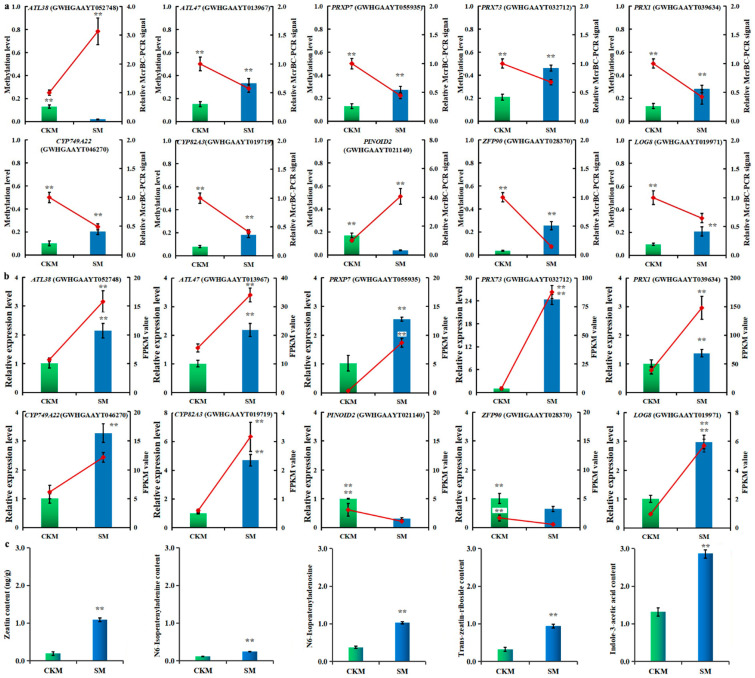
DNA methylation changes for the CHH sequence context are involved in cytokinin and auxin homeostasis in the two types of pear flower buds. (**a**) Methylation levels (MLs) of differentially expressed genes associated with differentially methylated regions (DMEGs) involved in the synthesis, transport, signal transduction, and catabolism pathways of cytokinin and auxin (in normal (CKM) and wizened (SM) flower buds). (**b**) Quantitative real−time PCR (qPCR) validation of relative expression levels of the genes described above. (**c**) Quantification of cytokinin and auxin concentrations in CKM and SM. Data (**a**–**c**) are presented as the mean ± standard deviation (SD). Vertical bars represent SD of the mean of at least three biological replicates. SPSS26 was used for statistical analysis. Student’s *t*-test was used for comparing two samples; asterisks indicate statistically significant differences between normal (CKM) and wizened (SM) flower buds (** means *p* < 0.01).

DEGs associated with the metabolism (biosynthesis or catabolism) of auxin and cytokinin in SM compared to CKM flower buds were chosen for further analysis. A total of seven hyper-DEGs and three hypo-DEGs were identified. Among them, the transcription of seven genes was upregulated and that of three genes was downregulated in SM relative to CKM (Figure 6a,b). It is interesting that expression of the four genes with the largest fold changes (more than threefold) was adjusted in the wizened flower buds in SM, including upregulated expression of *CYP749A22* (GWHGAAYT046270), *PRX73* (GWHGAAYT032712), and *CYP82A3* (GWHGAAYT019719) and downregulated expression of *PINOID2* (GWHGAAYT021140) (Figure 6a,b). Furthermore, these genes displayed a negative correlation between DNA methylation altered state and transcription change. Our results indicated that DNA methylation changes may play an important role in regulating gene expression once wizened flower buds have developed.

Previous studies have demonstrated that auxin and cytokinins take part in flower development, with unbalanced hormone metabolism causing flower buds to degenerate [8,9,17]. We found that the concentrations of the auxin indole-3-acetic acid (IAA) and the cytokinins N6-isopentenyladenine (IP), N6-isopentenyladenosine (IPR), *trans*−zeatin riboside (TZR), and zeatin increased once wizening happened in pear flower buds (Figure 6c). Then, we focused on DMEGs involved in the “auxin and cytokinin biosynthesis and metabolism processes” in SM compared to CKM flower buds. A total of ten DMEGs were enriched in these pathways, among which eight were shown to be hypermethylated and two to be hypomethylated in SM (Figure 6a and Supplementary Materials, Table S4).

**Figure 7:**
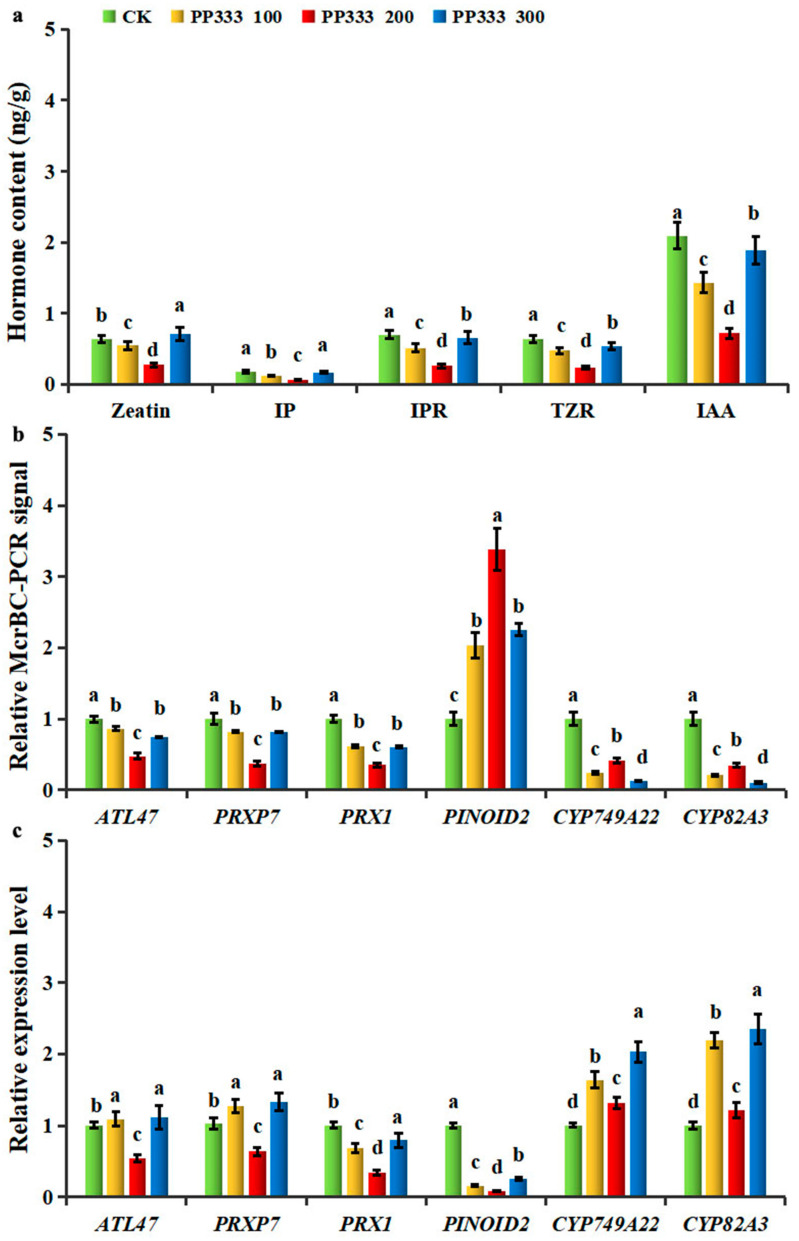
Exogenous paclobutrazol (PP333) treatment affected gene expression, methylation level, and hormone concentration of pear flower buds. (**a**) Relative endonuclease McrBC−PCR signal of differentially expressed genes associated with differentially methylated regions (DMEGs) involved in the synthesis, transport, signal transduction, and catabolism pathways of cytokinins and auxin after spraying with different concentrations of PP333. (**b**) Real-time quantitative PCR (qPCR) validation of relative expression levels of the genes after applying different concentrations of paclobutrazol (PP333). (**c**) Cytokinin and auxin concentrations after applying different concentrations of PP333. CK, PP333 100, PP333 200, and PP333 300 represent four treatment groups which were sprayed with 1000 mLof 0, 100, 200, 300 mg·L^−1^ paclobutrazol (PP333) in water every 15 days, respectively. Gene IDs: *ATL47* (GWHGAAYT013967), *PRXP7* (GWHGAAYT055935), *PRX1* (GWHGAAYT039634), *PINOID2* (GWHGAAYT021140), *CYP749A22* (GWHGAAYT046270) and *CYP82A3* (GWHGAAYT019719). Hormone abbreviations: IP, N6-isopentenyladenine; IPR, N6-isopentenyladenosine; TZR, *trans*−zeatin riboside; IAA, indole-3-acetic acid. Vertical bars represent standard deviations (SDs) of the mean of at least three biological replicates. Data (**a**–**c**) are presented as the means ± SDs. Values with different lowercase letters are considered significantly different between normal (CKM) and wizened (SM) flower buds (*p* < 0.05, a one-way ANOVA analysis of variance followed by Duncan’s multiple range test using SPSS26).

To investigate the influence of auxin and cytokinin on the generation of wizened flower buds, the effect of spraying exogenous paclobutrazol (PP333), a gibberellin biosynthesis inhibitor, onto pear flower buds was investigated. This hormone has been shown to affect cytokinin and auxin formation in fruit trees [18]. Application of 100, 200, or 300 mg·L^−1^ PP333 reduced the incidence of wizened flower buds to 46.2%, 20.1%, or 42.4%, whereas the control group, sprayed with H_2_O, exhibited a probability of wizening of 50.2%. At the same time, the concentrations of IAA, IP, IPR, TZR, and zeatin decreased after PP333 was applied to pear flower buds (Figure 7a). Application of 200 mg·L^−1^ PP333, which achieved the greatest decrease in the incidence of wizened flower buds, effectively reduced the wizening-related accumulation of both auxin and cytokinins.

To reveal the relationships between changes in DNA methylation and gene expression, we tested the relative McrBC-PCR signal and transcript levels in response to the PP333 application of the six genes involved in auxin and cytokinin metabolism identified earlier to show methylation differences between CKM and SM (Figure 7). Interestingly, all six DEGs associated with auxin and cytokinin metabolism which changed expression in response to the PP333 application also changed their DNA methylation status in different gene regions after the PP333 treatment. Except for *PINOID2* (GWHGAAYT021140), the other genes were hypomethylated in response to PP333 treatment (Figure 7b). In addition, these six DMEGs also regulated their expression levels in response to PP333 application (Figure 7c). In the case of the optimal concentration of 200 mg·L^−1^ PP333, *PINOID2* (GWHGAAYT021140), *CYP749A22* (GWHGAAYT046270), and *CYP82A3* (GWHGAAYT019719) displayed a negative correlation between DNA methylation level differences and transcription level changes, whereas the other three DEGs showed the opposite trend. Our results here confirmed that DNA methylation may have a positive regulatory role in the expression of many genes involved in the “auxin and cytokinin biosynthesis or metabolism” process in pear flower buds. Therefore, epigenetic regulation may play a significant role in maintaining the hormone balance during the normal development of flower buds.

## 3. Discussion

### 3.1. Mapping of the DNA Methylome in Pear Flower Buds by WGBS

DNA methylation has recently been recognized as a regulator of plant growth, development, and reproduction [19,20,21]. Some researchers have reported that dynamic changes in DNA methylation are involved in the regulation of flower formation in apple [22], *Arabidopsis* [23], azalea [24], basket willow [25], and chrysanthemum [26]. However, the genome-wide cytosine methylation profile and the possible role of DNA methylation in pear flower buds have not been reported to date. As a powerful technology used predominantly for DNA methylation research, whole-genome bisulfite sequencing (WGBS) can determine methylation patterns at single-base levels of resolution [27]. Recently, WGBS has been used to decrypt an increasing number of plant methylomes, ranging from model plants like *Arabidopsis* [28] and rice [29] to economically important fruits such as apple [22] and grape [30]. In the current study, the global DNA methylation pattern was profiled by WGBS in wizened flower SM buds and in normal CKM flower buds, both from the pear ‘Sucui 1’.

This is the first single-base-resolution DNA methylome constructed in pear buds and used to study the epigenetic regulation mechanism of flower bud formation. The genome-wide DNA methylation pattern in pear flower buds was found to be similar to, though slightly different from, a recent methylome analysis carried out by this research group on roots in a different pear species (*Pyrus betulaefolia*) during the growing season [12]. Specifically, in the earlier study, we observed higher levels of both CG (49.3–47.4%) and CHG (33.2–35.3%) methylation in pear flower buds (Figure 2c), relative to roots (34.0–39.9% for ^m^CG and 19.2–22.7% for ^m^CHG); meanwhile, the MLs in the CHH sequence context in pear flower buds (11.9–12.9%) were also greater than these in pear roots (3.9–4.7%) [12]. This difference to the results from the current study may have been due to the different pear species and tissues used in these two studies.

### 3.2. Changes in DNA Methylation and Their Association with Various Gene Expression Changes as Buds Wizened

Fewer ^m^Cs but higher MLs were observed among the three sequence contexts in SM compared with CKM (Figure 2a–c). However, the higher DNA methylation observed in SM buds, relative to CKM buds, may be due to the higher MLs of the existing ^m^Cs. Further analyses of DMCs showed that the increase in DNA methylation was mainly in the CG context (Figure 3b). All of the above analyses indicated that CG was significantly hypomethylated in SM relative to CKM buds. During the development of the flower buds of the model plant *Arabidopsis* from early to late stages, CG hypermethylation is often accompanied by local CHG and CHH hypomethylation [23]. On the other hand, CHH hypermethylation during rice ovule development and ripening is accompanied by CG and CHG hypomethylation [29]. However, the mean methylation levels were highest in the CG sequence context, followed closely by CHG and were lowest in the CHH context in all flower bud types of apple [22]. Our results found that CHH hypermethylation at the chromosome level may also be accompanied by increased methylation levels in CG and CHG once flower buds wizened (Supplementary Materials, Figure S1). These results indicate that the dynamic regulation of DNA methylation is critical for normal flower development, although DNA methylation changes in opposite directions in different plant species.

There was no significant difference in the number of hypermethylated (582) and hypomethylated (503) regions between SM and CKM buds (Figure 3c and Supplementary Materials, Figure S2a), a finding which was dissimilar to the DNA methylation pattern previously reported between different types of apple flower buds [22]. Consistent with previous studies on flower buds of apple [22], more DMR-related genes in SM were found to be CHH hypermethylated or hypomethylated in most gene regions in ‘Sucui 1’ (Figure 3c,d). The relationship between DNA methylation dynamics and gene expression can be revealed through the comparative integrative analysis of methylomes and transcriptomes [31]. DNA methylation is commonly considered to be a marker for transcriptional suppression. Indeed, combined genome-scale transcriptomic evidence in apple revealed that most methylation variances in the gene body or promoter regions have negative or positive influences on the expression profiles of genes, respectively [22]. In our study, some genes affected by DNA methylation were differentially expressed between SM and CKM buds, of which 76 genes were downregulated and 104 genes were upregulated (Figure 4b). This finding suggests an important role for DNA methylation in not only repressing gene expression but also in the activation of some genes during pear flower development.

### 3.3. Possible Regulatory Role of DNA Methylation in Pear Flower Buds

The ability to reproduce is an important feature of living organisms. Floral organs are the reproductive structures necessary to ensure the successful reproduction of angiosperms. It is well known that different hormones play various roles during flower organ development [8,9,17]. Usually, the disruption of auxin biosynthesis, polar auxin transport, or auxin signaling lead to the failure of flower formation [9]. The interaction between cytokinin and auxin during generative organ development is critical for the formation of flowers [32]. In the current study, we found that the concentrations of cytokinins and auxin in wizened buds were several times higher than those in normal buds (Figure 6c). This may be the main factor causing stamen degeneration and the inhibition of flower organ development in wizened buds.

Genome-scale transcriptomic evidence has revealed that the differentially expressed genes are the most over-represented in phytohormone signal transduction pathways when flower organs are initiated [31]. The results from our transcriptome analysis also found that hypo-DEGs in SM versus CKM buds (Figure 5b and Supplementary Materials, Table S3) were significantly enriched in the “plant hormone signal transduction” pathway. Interestingly, seven hypomethylated genes, which were all significantly upregulated in SM buds (Figure 6a,b), were involved in auxin sensing and signal transduction, auxin polar transport, cytokinin synthesis, and so on. These results indicate that the DNA hypomethylation that mediates auxin and cytokinin homeostasis may play a crucial role in pear flower bud development. In fact, epigenetic regulation may operate during flowering through hormone action [33]. For example, DNA methylation and gene expression in apple buds with different flowering capabilities suggest an epigenetic regulatory mechanism influencing apple flower bud formation following endogenous hormone changes [22]. Similar phenomena were also observed in the willow *Salix viminalis* [25].

In our study, PP333 treatment was carried out to reveal whether DNA methylation achieved changes in gene expression in response to altered hormone action. Spraying PP333 effectively reduced the occurrence of wizened buds by significantly decreasing the concentrations of cytokinins and auxin in buds. Indeed, application of paclobutrazol to other plants can affect the presence of authentic hormones, such as GA_3_, IAA, and zeatin [18,34,35]. The reason could be phytohormone crosstalk, through which the GA signal interacts with other hormonal signals including auxin and cytokinins and triggers downstream signaling cascades leading to metabolism, and so on, finally altering the latter’s contents [35]. For example, exogenous paclobutrazol affects the expression levels of genes involved in auxin and cytokinin metabolism [36]. In pear, several genes changed their DNA methylation levels at different gene regions, as well as changing their expression levels during this process. The relationship between DNA methylation and gene expression displayed either a negative or positive correlation. But, for a specific gene, how the change in gene DNA methylation level regulates its transcription level still needs to be confirmed by transgenic experiments. On the basis of previously published results and our own findings, we speculate that disturbed DNA methylation in SM buds may disrupt the balance between cytokinin and auxin levels in flower buds by inducing the expression of genes involved in auxin and cytokinin biosynthesis, polar auxin transport, or auxin signaling. This results in a burst of auxin and cytokinin that switches on stamen abortion and degeneration in SM buds, eventually leading to the formation of wizened flower buds. Controlling the accumulation of auxin and cytokinins, such as by PP333 application, can effectively alleviate flower bud abortion and wizening. However, the exact epigenetic regulatory network of hormone balance during flower bud development in the pear cultivar ‘Sucui 1’ remains a mystery. More experimental evidence is still needed to reveal how DNA methylation regulates the expression of specific genes involved in the hormone signaling pathway during flower bud development.

## 4. Materials and Methods

### 4.1. Plant Materials

The sand pear (*Pyrus pyrifolia*) variety ‘Sucui 1’ was selected from the hybrid offspring of the cross between ‘Huasu’ (♂) × ‘Cuiguan’ (♀) in 2011. The plants of ‘Sucui 1’, grafted onto Callery pear (*Pyrus calleryana)* rootstock, were planted in the spring of 2014 and managed, following the recommendations of local fruit growers, at Lishui Plant Science Base, Jiangsu Academy of Agricultural Sciences, Nanjing, Jiangsu Province, China (32°28′ N, 118°37′ E). The axillary buds of the current year’s branches were observed weekly from May to October 2022. We found that the wizened buds first occurred at the beginning of September. After two weeks, the proportion of the total buds on the current year’s growth that were wizened was about 50%. Representative growth branches were selected, cut, and brought back to the laboratory for morphological analysis on September 15th. The third buds from the tip of each branch were divided into normal flower buds (CKM) or wizened flower buds (SM). After removing their scales, 30 of the CKM and SM buds were kept in 70% formaldehyde–acetic acid–ethanol–distilled water (10:5:50:35) fixative (FAA) in preparation for paraffin sectioning. Other flower buds were snap-frozen in liquid nitrogen, and then stored at −80 °C prior to further analysis.

In 2023, the exogenous paclobutrazol application experiment was performed from June 15th to August 31st. Three hundred branches representing the current year’s growth were divided at random into four groups (75 branches per group) and sprayed with a total of 1000 mL of 0 (control), 100, 200, or 300 mg·L^−1^ paclobutrazol (PP333) in purified water every 15 days. On September 15th, the branches sprayed with each of the four treatments were harvested and brought to the laboratory, and the proportion of the total buds that were wizened was determined. The third bud from the tip of each branch was used as experimental material for hormone assays, gene expression, and methylation level detection studies.

### 4.2. Branch Scanning and Paraffin Section

The current year’s branches were stripped of leaves and petioles, then placed on the Microtek digital scanner (3700W; Shanghai, China) to collect images of CKM and SM buds. The internal morphologies of different flower buds were observed by the paraffin section method [37]. The paraffin block containing flower buds was section with a Leica RM2245 semi-motorized rotary microtome (Weztlar, Germany) and dyed with 1% toluidine blue. After neutral gum sealing, the sheets were observed and photographed under an Olympus BX53 optical microscope (Hachioji, Japan).

### 4.3. DNA Methylation Sequencing and Data Analysis

Total genomic DNA was extracted from pear flower buds (SM and CKM) with the DNeasy Plant Mini Kit (Qiagen, Hilden, Germany), following the manufacturer’s protocol. The DNA samples were purified by the MinElute PCR Cleanup Kit (Qiagen). The DNA integrity and purity were determined via electrophoresis on 1% (*w*/*v*) agarose gels and by UV spectrophotometry with a BioPhotometer Plus (Eppendorf, Hamburg, Germany). After mechanical degradation into double-strand breaks by ultrasonic treatment, terminal repair and 3′ terminal addition of A bases were carried out [12], and 1 μg of genomic DNA was bisulfite-converted using an EZ DNA Methylation-Gold™ kit (Zymo Research, Irvine, CA, USA). Finally, whole-genome bisulfite sequencing (WGBS) was conducted on the Illumina NovaSeq 6000 platform (Genepioneer, Nanjing, China), and 150 bp paired-end reads were generated. The two samples, namely wizened and normal flower buds, were each represented by three biological replicates. Trim fastp (v0.20.0) was used to quality control, filter low quality readings, and remove adapter sequences [38]. Then, Bismark (v0.24.0) and bowtie2 (v2) [39] were used to compare the high-quality pruned read segment with the pear reference genome (https://www.ncbi.nlm.nih.gov/datasets/genome/GCA_007844245.1/) accessed on 7 December 2022 [15]. Bismark (command -no_overlap) was used to identify methylated cytosine in comparison readings. Differentially methylated regions (DMRs) in the three cytosine sequence contexts (CG, CHG, and CHH) were then identified using swDMR [40]. Windows with more than four informative cytosines (i.e., read coverage ≥ 4), absolute methylation differences of more than 0.15 (for C), 0.20 (for CG), 0.15 (for CHG), or 0.10 (for CHH), and a Benjamini–Hochberg-adjusted false discovery rate (FDR) < 0.05 (Fisher’s exact probability test) were considered to be DMRs. DMRs separated by gaps of less than 100 bp were merged, and the final coordinates of each DMR were adjusted from the first methylated cytosine to the last methylated cytosine [41]. The genetic characterization of DMR was annotated using Bedtools (v2.21.0) [42].

### 4.4. RNA-Seq

Total RNA from CKM and SM flower buds was isolated and purified using TRIzol reagents (Invitrogen, Carlsbad, CA, USA). The total RNA was confirmed to be >100 ng·μL^−1^, with an OD_260_/_280_ > 1.8 and an RIN number > 7.0, using a BioPhotometer Plus (Eppendorf, Hamburg, Germany) and an Agilent Bioanalyzer 2100 (Santa Clara, CA, USA). An aliquot of 20 μg total RNA was used to construct a transcriptomic library using the NEBNext^®^ Ultra™ RNA Library Prep Kit for Illumina^®^ (NEB, Ipswich, MA, USA). Finally, RNA-Seq was conducted on the Illumina NovaSeq 6000 platform (Genepioneer, Nanjing, China), and 150 bp paired-end reads were generated. The two samples, namely wizened and normal flower buds, were each represented by three biological replicates. The FastQC quality control analytical tool (http://www.bioinformatics.babraham.ac.uk/projects/fastqc/) accessed on 7 December 2022 and was chosen as the read quality control indicators, and Trim fastp (v0.20.0) was used to remove adapter sequences and low-quality sequences from the raw data [38].

The clean reads obtained from the filtered transcriptome sequencing data were compared with the pear genome [15] using the HISAT alignment program (v2.1.0, default parameters) [43]. Fragments per kilobases per million reads (FPKM) values were calculated by comparing the results to form BAM files. The differentially expressed genes among the samples were identified by the R package ‘DESeq2’ [44], and |log2 fold change| > 1 and FDR < 0.05 were used as the criteria for statistical analysis and screening. The differential genes obtained were identified by BLAST at the COG (Clusters of orthologous groups of proteins), GO (Gene Ontology), KEGG (Kyoto Encyclopedia of Genes and Genomes), Nr (NCBI Non-redundant Proteins), Pfam (the Pfam protein families database), and Swiss-Prot (Swiss-Prot protein sequence) databases. Protein function annotation and classification statistics were carried out.

### 4.5. Combined Transcriptome and Methylome Analysis

The DMR region from the WGBS analysis was annotated using Bedtools (v2.21.0) [42]. The Perl program language was used to obtain differentially methylated genes (DMG) for analysis, which were at the intersection of the transcriptome result and the differentially methylated region (DMR) result at the specified location. Finally, the differential gene members contained in DMG were identified from the pear genome [15].

### 4.6. McrBC-PCR and Quantitative Real-Time PCR (qPCR)

The fluorescent qPCR gene-specific primers were designed using Primer Premier 5.0 (Table S5) and synthesized by Sangon Biotech (Shanghai, China). All primers were tested by PCR amplification, electrophoresis, and dissolution curves to ensure primer specificity. A LightCycler^®^ 480 II (Roche, Basel, Switzerland) was used for qPCR, and the reaction was performed according to the instructions for the Genious 2 × SYBR Green Fast qPCR Mix (ABclonal, Wuhan, China). The relative expression of the housekeeping gene *PpActin* was calculated using the 2^−ΔΔCT^ formula. The endonuclease McrBC (M0272S, NEB) was used to digest 1 μg of genomic DNA from pear buds according to the manufacturer’s instructions. Digestion reactions without GTP were used as negative controls for standardized analysis. Methylated DNA could be digested by McrBC, and PCR results showed that the qPCR signal level was negatively correlated with the methylation level [45]. The design and synthesis of McrBC-PCR primers were the same as for qPCR analysis, and the primer sequences are shown in Table S6.

### 4.7. Hormone Assays

The endogenous auxin and cytokinins in flower buds were extracted and their concentrations were determined according to a method used by Pan et al. [46]. After grinding, a 0.5 g sample of homogenate was added to 10 mL of acetonitrile with 8 μL of an internal standard. Following extraction at 4 °C overnight and centrifugation at 12,000× *g* for 5 min at 4 °C, the supernatant was obtained. To the pellet another 5 mL aliquot of acetonitrile was added, and the pellet was extracted twice more with all the supernatants combined, to which C18 and GCB (Anpel, Shanghai, China) were added to remove impurities, followed by centrifuging at 4 °C at 12,000× *g* for 5 min. The resulting supernatant was dried under nitrogen, the residue redissolved in 400 μL methanol, passed through a 0.22 μm organic phase filter membrane, and stored at −20 °C.

High-performance liquid chromatography (HPLC) was carried out on an Agilent 1290 HPLC (Santa Clara, CA, USA), and the detector was the SCIEX-6500Qtrap (tandem mass spectrometer, MS/MS) (Redwood City, CA, USA). Chromatography was performed on a Poroshell 120 SB-C18 reverse-phase column (2.1 i.d. × 150 mm, 2.7 µm particle diameter, Agilent). The mobile phase A solvent was methanol with 0.1% formic acid, and the mobile phase B solvent was water with 0.1% formic acid. The flow rate was 0.3 mL min^−1^. The column temperature box was maintained at 30 °C and the injection volume was 2 µL each time. Retention times were 3.63, 3.83, 4.63, 4.84, and 5.53 min for zeatin, *trans*-zeatin riboside (TZR), N6-isopentenyladenine (IP), N6-isopentenyladenosine (IPR), and indole-3-acetic acid (IAA), respectively. Finally, standard curves were used to calculate hormone concentrations. Standard products IAA, IP, IPR, TZR, and zeatin were purchased from Sigma (St. Louis, MO, USA), and HPLC grade methanol and acetonitrile were purchased from Merck (Darmstadt, Germany).

### 4.8. Statistical Analysis

SPSS26 (IBM Corp., Armonk, Chicago, IL, USA) was used for our statistical analysis. Student’s *t*-test for comparing two samples; ANOVA and Duncan’s multiple range test for more than two samples, and Pearson’s correlation coefficient test to study relationships. *p* < 0.05 (*) and *p* < 0.01 (**).

## 5. Conclusions

Through integrated methylome and transcriptome analysis, a total of 1084 DMRs and 553 DMGs were identified in a comparison of the wizened SM pear flower buds and the normal CKM flower buds. Among the DMEGs, 103 genes were upregulated and 75 genes were downregulated. The hypo-DEGs were significantly enriched in the “plant hormone signal transduction” pathway, and these hypomethylated genes (involving genes for auxin sensing and signal transduction, auxin polar transport, and cytokinin synthesis) were all significantly upregulated in SM buds. Combined with our biochemical data, we propose that DNA hypomethylation is involved in regulating the homeostasis of auxin production and cytokinin synthesis to maintain the normal development of pear flower buds. Our results help us to better understand the possible role of specific gene DNA methylation in pear flower bud development and will accelerate the study of the molecular mechanisms of flower bud development in pear.

## Data Availability

MDPI Research Data Policies at https://www.ncbi.nlm.nih.gov/sra/SRR24058259 to SRR24058264 accessed on 7 April 2023.

## Supplementary Materials

The supporting information can be downloaded at: https://www.mdpi.com/article/10.3390/ijms25137180/s1.
