# Supplementary material for: Integrated Methylome and Transcriptome Analysis between Wizened and Normal Flower Buds in Pyrus pyrifolia Cultivar ‘Sucui 1’
Source: Int J Mol Sci. 2024 Jun 29;25(13):7180. doi: 10.3390/ijms25137180 (PMC11241763; doi:10.3390/ijms25137180)
Supplement: Supplementary file 1 [file ijms-25-07180-s001.zip › 5. Supplementary materials.pdf]

Supplementary materials

Table S1. Output data description of whole-genome bisulfite sequencing (WGBS) for control (CKM) and wizened flower buds (SM)

Table S2. Gene Ontology (GO) enrichment analysis of hyper- or hypomethylated differentially expressed genes (DEGs) for the CHH sequence context in control (CKM) versus wizened flower buds (SM)

Table S3. Kyoto Encyclopedia of Genes and Genomes (KEGG) pathway enrichment analysis of hyper- or hypomethylated differentially expressed genes (DEGs) for the CHH sequence context in control (CKM) versus wizened flower buds (SM)

Table S4. Information on ten differentially expressed genes associated with differentially methylated regions (DMEGs) in the plant hormone pathways involved in the wizened flower buds of pear

Table S5. Primers used for real-time quantitative PCR (qPCR) in this study

Table S6. Primers used for MrBC-PCR in this study

Figure S1. Levels of C, CG, CHG, and CHH methylation in the 17 pear chromosomes.

The X-axis represents all 17 chromosomes of pear and the Y-axis represents methylation levels (MLs) in different sequence contexts.

Figure S2. Volcano map of identified differentially methylated regions (DMRs) of C-sites (a) and their length distribution (b) in control (CKM) versus wizened flower buds (SM).

In the volcano map, each dot signifies one DMR. Among them, the red and blue dots represent hyper- and hypomethylated DMRs, respectively. The X-axis represents the methylation level (ML) difference and the Y-axis represents the opposite number of

log_{10} value of the *P*-value.

Figure S3. Evaluation of RNA-sequencing data quality. (a), (c) and (e) show Pearson's correlation coefficient analysis of the control flower bud (CKM) dataset. (b), (d) and (f) show Pearson's correlation coefficient analysis of the wizened flower bud (S) dataset.

Figure S4. Numbers of differentially expressed genes (DEGs) in control (CKM) versus wizened flower buds (SM).
The X-axis is the log_2 value of fold change and the Y-axis is the opposite number of log_{10} value of the FDR (false discovery rate, adjusted *P*-value). Each dot represents one gene. Among them, the green and red dots represent up- and down-regulated genes, respectively, and the blue dots represent genes without significantly differential expression.

Figure S5. Comparative analysis of methylation densities of C-sites in different gene regions of the up- (a) or downregulated (b) differentially expressed genes (DEGs), including promoter, exon and intron regions.

Figure S6. Gene Ontology (GO) enrichment analysis of hyper- (a) or hypomethylated (b) differentially expressed genes (DEGs) for the CHH sequence context in control (CKM) versus wizened flower buds (SM).
The bars followed by an asterisk indicate significantly enriched GO terms
