# Supplementary figures and images for: Integrated Methylome and Transcriptome Analysis between Wizened and Normal Flower Buds in Pyrus pyrifolia Cultivar ‘Sucui 1’
Source: Int J Mol Sci. 2024 Jun 29;25(13):7180. doi: 10.3390/ijms25137180 (PMC11241763; doi:10.3390/ijms25137180)

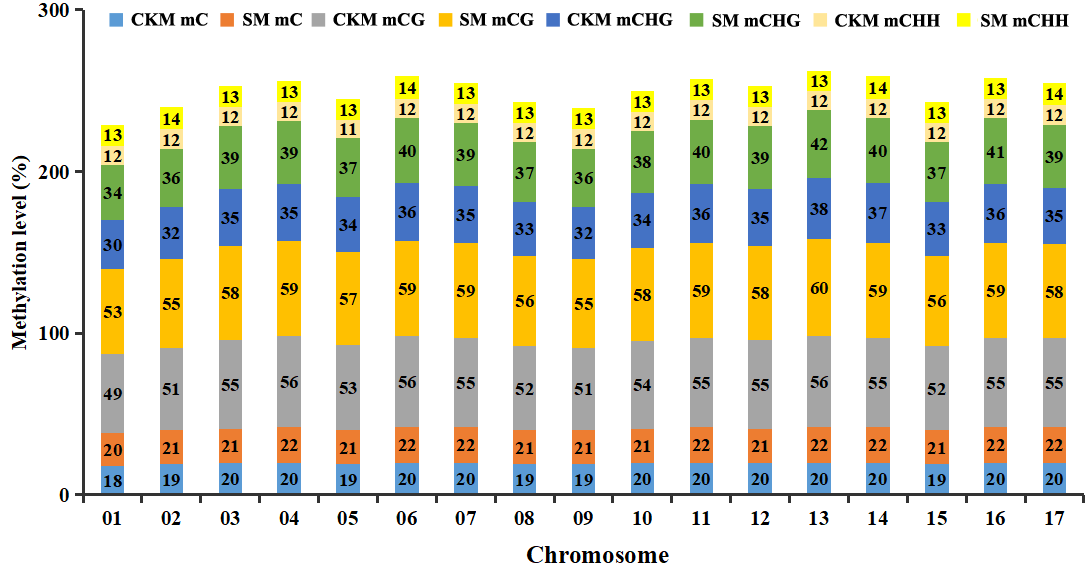

Supplement: Supplementary file 1 [file ijms-25-07180-s001.zip › FigureS1.tif]

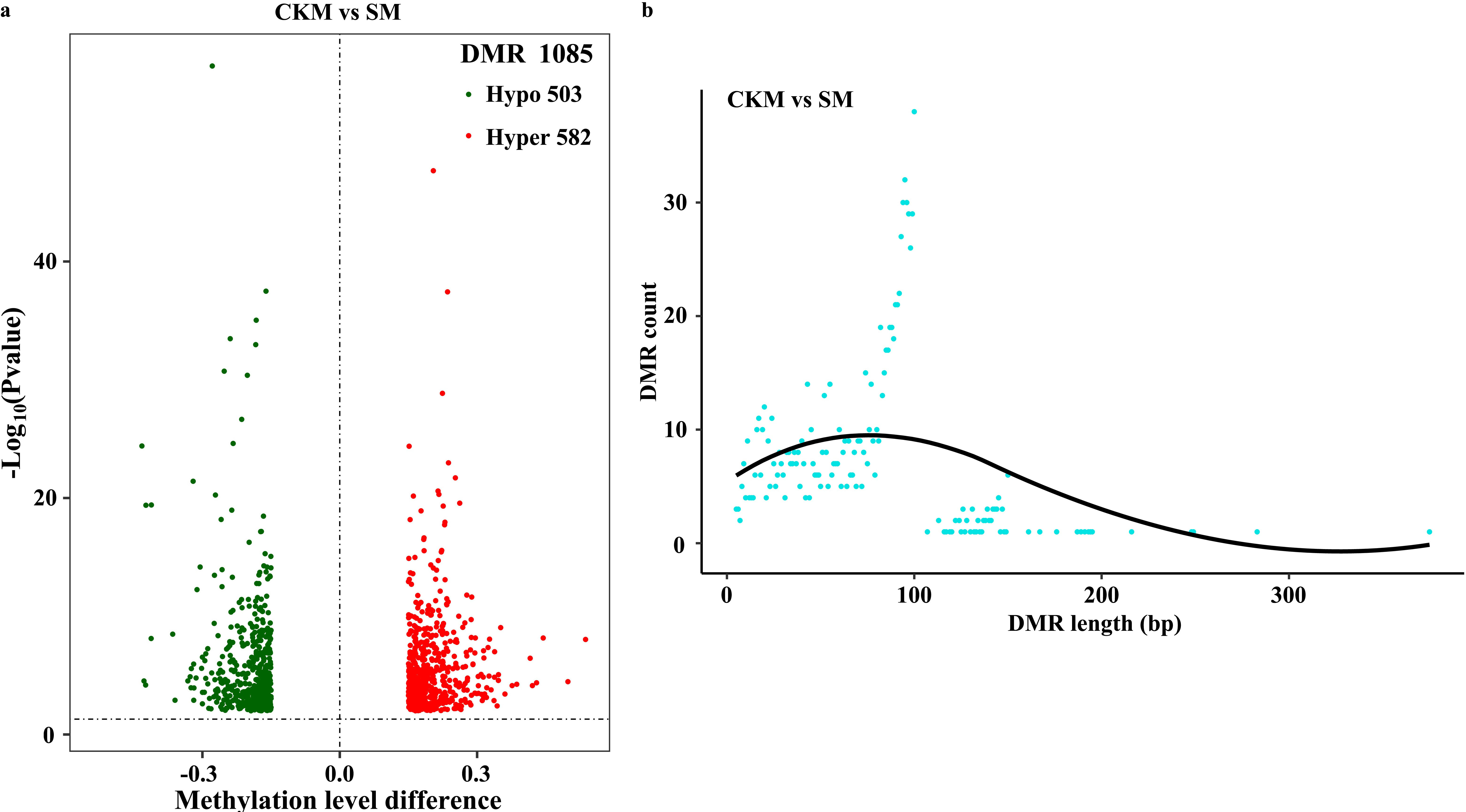

Supplement: Supplementary file 1 [file ijms-25-07180-s001.zip › FigureS2.tif]

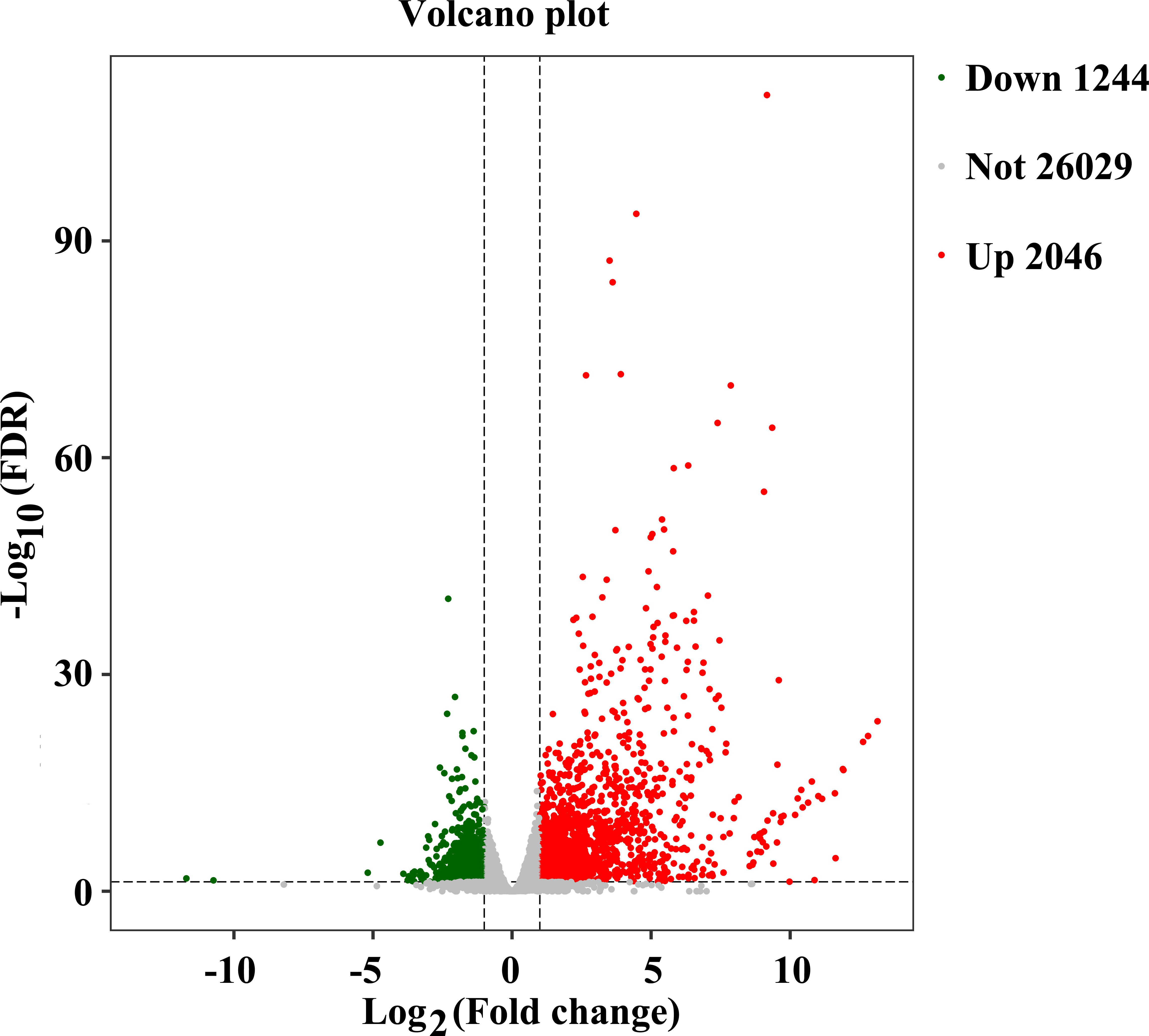

Supplement: Supplementary file 1 [file ijms-25-07180-s001.zip › FigureS4.tif]

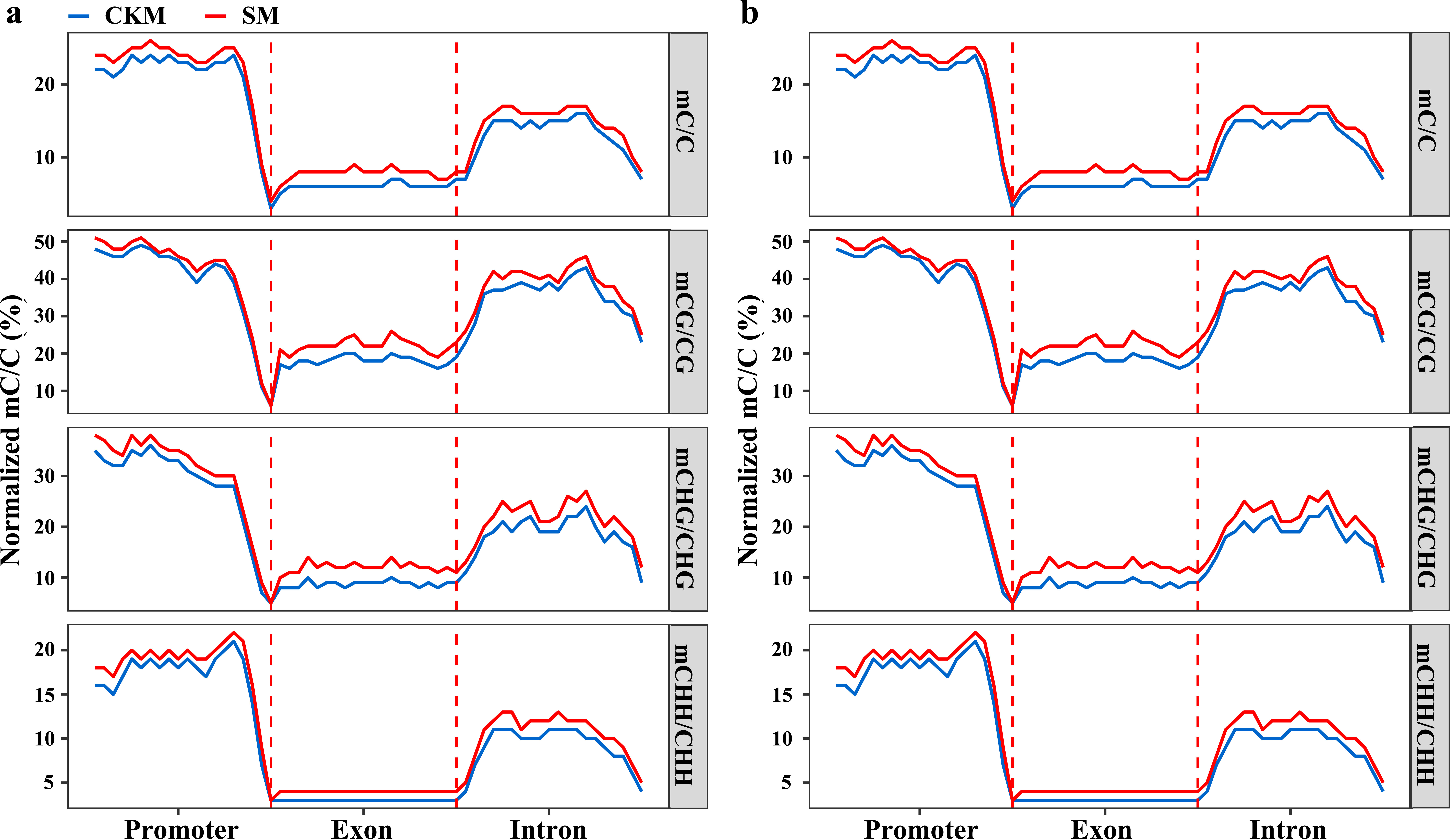

Supplement: Supplementary file 1 [file ijms-25-07180-s001.zip › FigureS5.tif]
